# Global wastewater microbiome reveals core bacterial community and viral diversity with regional antibiotic resistance patterns

**DOI:** 10.1128/msystems.01428-24

**Published:** 2025-09-17

**Authors:** Yueyang Yan, Xiaoyan Zhao, Xingxing Liang, Ying Xue, Qichen Niu, Dong Li, Xianqi Zhou, Yaoming Li, Shikui Dong, Yunpeng Gai

**Affiliations:** 1 School of Grassland Science, Beijing Forestry University 12380, Beijing, China; 2 School of Management, Shanxi Medical University, Taiyuan, China; CNRS Delegation Alpes, Lyon, Rhône-Alpes, France

**Keywords:** wastewater microbial communities, antibiotic resistance genes, pathogen transmission, urbanization, industrialization, metagenome-assembled genomes, biogeographical distribution, wastewater treatment plants, public health, environmental sustainability

## Abstract

Municipal wastewater treatment plants (WWTPs) serve as global repositories for diverse and dynamic microbial communities, reflecting the complex interplay of human activities, environmental conditions, and public health challenges. Despite their importance, a comprehensive understanding of the global distribution, composition, and functional roles of these microbial ecosystems has remained elusive. Here, we present a comprehensive analysis of bacterial and viral diversities in global wastewater systems by examining 575 sampling sites across 74 cities in 60 countries. Through metagenomic analysis, we reconstructed 12,758 non-redundant bacterial metagenome-assembled genomes (MAGs) spanning 70 phyla, with 4,499 MAGs representing novel species. Despite considerable regional variation, we identified a consistent core microbiome present across 70% of global samples predominantly comprising Proteobacteria. We further assembled 1.7 million viral genomes, revealing unprecedented viral diversity with over 1.5 million species-level viral operational taxonomic units (vOTUs). Network analysis demonstrated that transport proteins play crucial roles in maintaining WWTP functional resilience against disturbances. Machine learning approaches effectively predicted continental origins of wastewater samples based on microbial signatures, confirming that microbial communities reflect local environmental and socioeconomic conditions while maintaining functional conservation. We observed significant variation in the antibiotic resistance gene (ARG) distribution, with elevated prevalence in certain African and Asian regions compared to Europe and North America. Our results establish wastewater microbiomes as important indicators of human activity and provide critical insights for advancing environmental monitoring, antimicrobial resistance surveillance, and wastewater-based epidemiology.

**IMPORTANCE:**

Intensifying urbanization and human activities have dramatically increased global wastewater generation, creating complex microbial ecosystems that significantly impact environmental and public health. This study presents the first large-scale, comprehensive characterization of bacterial and viral communities in wastewater treatment systems worldwide. By analyzing samples from diverse geographical, climatic, and socioeconomic contexts, we reveal how wastewater microbiomes serve as microbial fingerprints of human society, reflecting regional characteristics while maintaining functional conservation. Our findings demonstrate that these communities function as ecological extensions of human gut microbiota in the external environment, with important implications for the spread of antibiotic resistance and pathogens. The identification of viruses as key metabolic regulators in these systems provides new perspectives on microbial community dynamics. This global-scale analysis advances our understanding of wastewater microbiology and offers valuable insights for improving wastewater management, enhancing environmental monitoring systems, and strengthening public health surveillance through wastewater-based epidemiology.

## INTRODUCTION

The world generates an estimated 380 billion cubic meters of wastewater annually—equivalent to five times the annual flow of Niagara Falls—with projections indicating a 24% increase by 2030 and 51% by 2050 (1). This dramatic upward trend not only reflects intensified human activities and accelerated urbanization but also exerts multifaceted pressure on global environmental sustainability, public health security, and social infrastructure (2). As a crucial byproduct of urban metabolism, wastewater exhibits compositional complexity and diversity, originating from residential activities, industrial production, and agricultural operations. These wastewaters typically contain high concentrations of organic matter, nutrients, heavy metals, pathogenic microorganisms, and antibiotic residues (3). Without appropriate treatment prior to discharge, these pollutants infiltrate hydrological cycles, disrupt ecological equilibrium, contaminate drinking water sources, bioaccumulate through food chains, and ultimately pose severe threats to human health. Consequently, comprehensive understanding and effective management of wastewater microbial communities are considered pivotal issues in contemporary environmental science and public health (4).

In recent years, wastewater microbiology research has achieved significant progress across multiple urban centers globally, with particularly rich findings from densely populated regions, such as New York and Hong Kong (5, 6). These metropolitan environments provide ideal observational windows for examining how microbial communities adapt to unique ecological niches and their complex interaction patterns (7). While these studies have substantially deepened our understanding of microbial processes in wastewater treatment, they also highlight a critical knowledge gap—the absence of microbial metagenomic research at a global scale, which constitutes the theoretical foundation of our study. With the intensification of global urbanization, industrial development, and climate change, wastewater treatment plants (WWTPs) have gained increasing strategic importance in global environmental governance (8). These facilities transform wastewater contaminants into discharge-compliant gaseous, liquid, and solid phase substances through precisely integrated physical, chemical, and biological treatment processes, playing an irreplaceable role in safeguarding water resources and public health (9). Notably, continuous innovation in wastewater treatment technologies has not only enhanced pollutant removal efficiency but also pioneered new pathways for resource recovery and energy utilization, providing technical support for circular economy development (10). The microbial community within wastewater, which resembles that of the human gut, includes bacteria, viruses, fungi, and protozoa—all of which play critical roles in treatment processes (11).

Wastewater is recognized as a complex chemical and biological matrix, serving as a direct mirror of human activity (12). The presence of chemical substances and biomarkers in wastewater, along with their correlations to population size, consumption patterns, and environmental exposure, provides objective evidence for interpreting human behaviors within specific watersheds (13). For instance, pharmaceutical residues in wastewater can precisely reflect regional population health status and medication habits, while nutrient indicators can reveal trends in dietary structures and lifestyle changes (14). As microscopic indicators of human activity, wastewater microbial communities exhibit structural and functional characteristics that demonstrate significant co-variation with macroscopic factors, such as geographical environment, climatic conditions, disease prevalence, production activities, and lifestyle habits (15). These microbial communities display distinct regional specificity, with their diversity and functional distribution closely related to local climatic conditions, geographical environments, population density, and economic development levels (16). There is a complex, interconnected relationship between wastewater microbial communities and the human gut microbiota. The human gut microbiome comprising trillions of microorganisms serves as a core participant in host health maintenance, immune system regulation, and nutrient metabolism. Similarly, wastewater microbial communities can be viewed as ecological extensions of human gut microbiota in the external environment, becoming key drivers of pollutant degradation and transformation in wastewater treatment systems.

The widespread distribution of antibiotic resistance genes (ARGs) in wastewater systems exhibits high consistency with patterns observed in human gut microbiomes, strongly suggesting the risk of ARGs potentially spreading to the environment through wastewater systems (17). The viral communities in wastewater, particularly enteric viruses, can significantly alter bacterial community structures through infection and genetic recombination, thereby influencing the dynamic equilibrium of entire microbial communities. A thorough understanding of these complex relationships is crucial for designing targeted wastewater treatment strategies and effectively suppressing the spread of antimicrobial resistance and viral pathogens (18). This knowledge framework not only strengthens our management capabilities for wastewater systems but also provides valuable perspectives for exploring the profound connections between human health and environmental microbiomes (19). Furthermore, elucidating the linkages between microbial communities in wastewater treatment systems and the human gut microbiome will deepen our understanding of viral roles in gene exchange between microbes, microbial community regulation, and overall wastewater ecosystem function (20). Specifically, viruses in wastewater can facilitate horizontal transfer of antibiotic resistance genes among bacterial populations, potentially enhancing bacterial resistance. This genetic material exchange not only reshapes the structure and function of wastewater microbial communities but may also transmit resistance genes to humans through various pathways, constituting a significant public health concern. From an environmental management perspective, these microorganisms are essential for degrading and transforming pollutants, recovering nutrients and energy, and mitigating pathogens, thereby reducing disease transmission pathways (21). Their contributions extend to microbial ecology and urban sustainability by helping maintain ecosystem balance and enhancing resilience against environmental stressors in urban areas (22).

The systematic investigation of microbial communities in urban sewage systems carries profound scientific significance and practical value. In contemporary urban configurations, sewage systems function not merely as critical infrastructure for wastewater treatment but also as complex and dynamic microbial ecosystems. Previous research on urban environmental microbial communities has established a solid foundation for understanding the complex dynamics of microbial life in localized environments. In-depth exploration of specific microbial functions, such as denitrification, and their community structural composition has greatly enriched our understanding of wastewater treatment microbial processes and their environmental health impacts (23–25). However, these findings also highlight a key scientific proposition—the need to construct an integrative global perspective that transcends individual cities and specific microbial functions (26). To comprehensively decipher the multifaceted functions of wastewater microbiomes, future research must encompass diverse urban centers representing different population characteristics, geographical locations, and climatic conditions.

This study aims to systematically reveal the bacterial and viral diversities constituting the global wastewater microbiome and their ecological functions. Through a high-throughput analysis of metagenome-assembled genomes (MAGs) and viral data sets from 575 sampling sites across 74 cities in 60 countries, we successfully retrieved numerous viral and bacterial genomes from global wastewater treatment plant communities. This investigation represents the first large-scale, comprehensive analysis of wastewater treatment plant bacteria and viruses to date, providing a systematic perspective for deepening our understanding of global wastewater microbial diversity patterns, biogeographical distribution principles, and their critical roles in biogeochemical cycles.

## MATERIALS AND METHODS

### Sample collection

The data sets utilized in this study were retrieved from the European Nucleotide Archive (ENA) (27). Given the extensive scope of wastewater samples, metagenomics, and environmental microbiota surveillance, we developed a comprehensive search strategy to identify relevant data sets. Initially, we screened the ENA using specific keywords to identify potential data sets related to wastewater samples, metagenomics, and environmental microbiota. Subsequently, we filtered these data sets to include those with comprehensive metadata descriptions regarding wastewater sources, sampling sites, and sequencing technologies for further analysis.

Our study methodology incorporated meticulous data preparation through rigorous quality checks to ensure the integrity and high quality of the selected data sets. Sequence reads were trimmed using Trimmomatic v0.38 with the following parameters: LEADING:3 (removing bases at the 5′ end with quality scores below 3), TRAILING:3 (removing bases at the 3′ end with quality scores below 3), and SLIDINGWINDOW:4:15 (trimming sequences based on a sliding window of size 4, ensuring the average quality score within the window is not lower than 15). By combining samples from similar projects, we assessed common trends in sewage microbiota variability across diverse environmental conditions. This integrative approach was essential for developing a comprehensive understanding of the dynamics influencing the diversity and evolutionary characteristics of sewage microbiota.

### Metagenomic assembly and genome structure construction

We conducted metagenomic taxonomic classification to obtain an in-depth microbiome overview. For assembly, we employed MEGAHIT with parameters "-min-contig-len 500 -t 40" to generate maximum assembled overlapping clusters and individual assemblies for each sample (28). Binning was performed using three complementary methods: MaxBin, MetaBAT2, and CONCOCT (29–31). For each approach, default settings were determined based on sequence configuration and coverage depth. We assessed genome completeness and contamination using CheckM with the lineage_wf workflow to evaluate all 12,864 bins (32). Subsequently, dRep was utilized to dereplicate the MAGs at a 99% average nucleotide identity (ANI) threshold, yielding a set of non-redundant MAGs (33). Open reading frames (ORFs) were predicted using Prodigal with the parameter "-p single," and sample abundance was estimated using the "quant_bins" module of metaWRAP (34).

### Functional annotation

For functional annotation, we utilized the NCBI-NR, eggNOG (35), and UniProt Swiss-Prot databases. Relative gene abundance was determined by aligning high-quality reads against previously reported gene catalogs. Specifically, virulence factor gene (VFG) sequences and their corresponding protein databases were used to analyze pathogen functional genes (36). Mobile genetic element (MGE) profiles were generated by mapping reads to custom MGE databases based on previous literature (37).

### Machine learning

Our analytical approach incorporated the Jupyter Lab programming environment for data analysis and modeling (38). During data preprocessing, we employed the train_test_split() function from scikit-learn to partition the data set into training and test sets at a 70:30 ratio, ensuring effective model validation on independent test data. Based on fivefold cross-validation results, we selected LightGBM as the primary model algorithm. LightGBM founded on a decision tree gradient boosting framework significantly enhances computational efficiency when processing large-scale data and delivers superior performance across various machine learning tasks. To further strengthen model stability and generalization capacity, we implemented scikit-learn’s KFold for k-fold cross-validation. This methodological approach divides the data set into multiple subsets, ensuring consistent model performance across different combinations of training and validation data, effectively mitigating overfitting, and enhancing generalization capabilities.

### Network construction and analysis

We utilized the Hmisc package in R to calculate data correlations and construct a co-occurrence network visualized using the Fruchterman-Reingold algorithm in Gephi software. The network was subsequently partitioned into distinct subnetworks based on acquired regions. Hub genomes were identified by determining nodes with the highest connectedness in each network using the eigenvector centrality algorithm in Gephi.

### Viral genome assembly and annotation

To categorize generated fragments into viral genomes, we employed the VirSorter program, which utilizes machine learning techniques for classification while comparing assembled fragments with established viral and host genomes (39). This approach facilitated the identification and categorization of viral genomes present in wastewater treatment systems. CheckV was subsequently used to evaluate viral genome integrity and quantify contamination levels, enabling the assessment of the completeness and impurity of constructed viral genomes(40). Additionally, we performed a functional annotation of viral genomes through comparative analysis with known databases, enabling predictions and annotations regarding their functional characteristics.

### Host prediction

We conducted BLAST searches against phage subpopulations for expected CRISPR spacers using parameters (-evalue 1e-10, task "blastn-short") (41). To definitively confirm a bacterium as a host, we required at least two spacer matches with the phage, allowing for a maximum of one mismatch.

### Clustering of viral genomes into viral operational taxonomic units

Viral genomes were clustered into species-level viral operational taxonomic units (vOTUs) using criteria of 95% average nucleotide identity and an 85% sequence alignment score. To identify genus and family-level vOTUs, we integrated gene sharing and average amino acid identity methods. BLASTp alignment of all viral proteins was performed using DIAMOND software (42, 43).

## RESULTS

### Reconstruction of 12,758 MAGs from large-scale genome-resolved metagenomics

**Fig 1:**
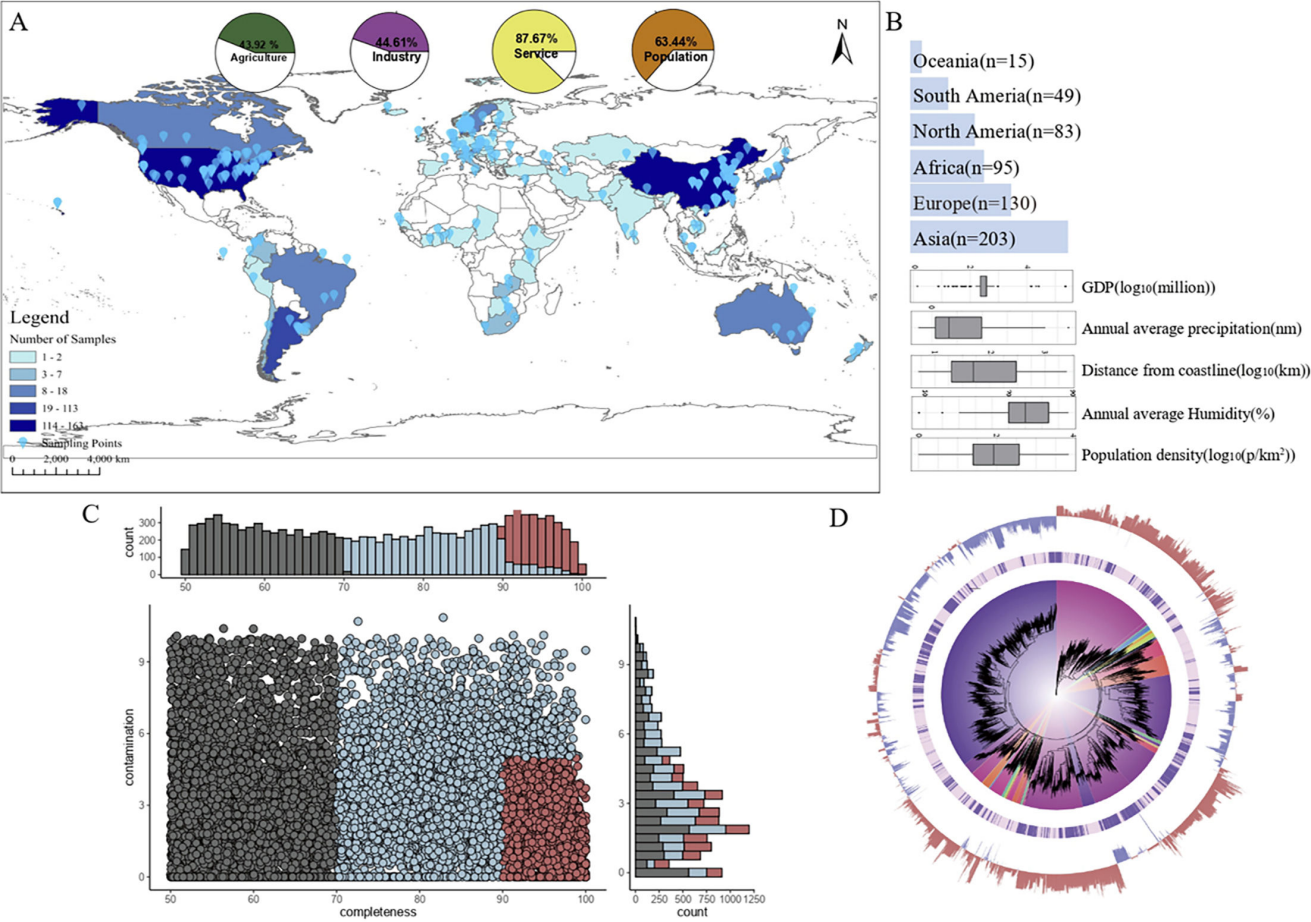
Global distribution and genomic characteristics of microbial communities in global wastewater. (**A**) Geographic distribution of the WWPT samples. Colors represent the number of samples collected at each location. The map was created by the authors using publicly available geographic data and open-source mapping tools. (**B**) The bar chart represents the total number of samples collected for each continent. The boxplot offers a comprehensive depiction of the samples, presenting the distribution of location-specific data on GDP, annual average precipitation, distance from the coastline, annual average humidity, and population density. (**C**) Completeness and contamination of 12,765 genomes of the WWPT microbiome. (**D**) The maximum-likelihood tree of the MAGs identified in this study was generated using PhyloPhlAn based on concatenated protein sequences. Clades are color-coded by phyla, and novelty annotations are displayed in the inner layers. The middle layer highlights novelty annotations, indicating MAGs that have not been previously reported (ANI > 95% with known genomes). The outermost frontier of the plot illustrates the GC content disparity of the MAGs, where red represents GC content higher than 50%, and blue indicates GC content lower than 50%.

To reconstruct bacterial and viral genomes, we analyzed a diverse array of sewage samples from 575 sampling sites across 74 cities in 60 countries, spanning six continental regions (Fig. 1A). We integrated geographical, meteorological, population density, economic, and demographic data for each sampling location. The samples originated from diverse climatic environments, including cold, temperate, humid, and arid regions, and encompassed nations at various stages of economic development, from industrialized regions to emerging economies (Fig. 1B).

The assembly of MAGs involved processing approximately 6.85 TB of metagenomic sequencing data, resulting in 12,875 metagenome-assembled genomes (MAGs). Each MAG exhibited a minimum completeness of 50% with contamination not exceeding 10%. Dereplication at a 99% average nucleotide identity threshold yielded 12,758 non-redundant MAGs. A subset of 2,556 high-quality MAGs demonstrated >90% completeness with an average contamination rate of 2.6% (Fig. 1C). These high-quality MAGs displayed remarkable diversity in genome size, ranging from 0.3 to 9 MB.

The taxonomic classification of the MAGs encompassed a broad spectrum of taxonomic groups, including 70 phyla, 121 classes, and 288 orders (Fig. 1D). Proteobacteria comprising 33.9% of WWTP bacteria play crucial roles in global carbon, nitrogen, phosphorus (44), and sulfur cycles (45). Bacteroidetes representing 13.89% are distinguished by their capacity to degrade proteins, lipids, and other macromolecules, significantly contributing to volatile fatty acid production (46). Patescibacteria constituting 5.49% of the bacterial population and commonly found in groundwater and surface water are instrumental in denitrification processes (47). The identification of 4,499 MAGs unclassified at the species level highlights the novelty and diversity of microbial species uncovered in this investigation.

### A core wastewater microbiome centers on global diversity

**Fig 2:**
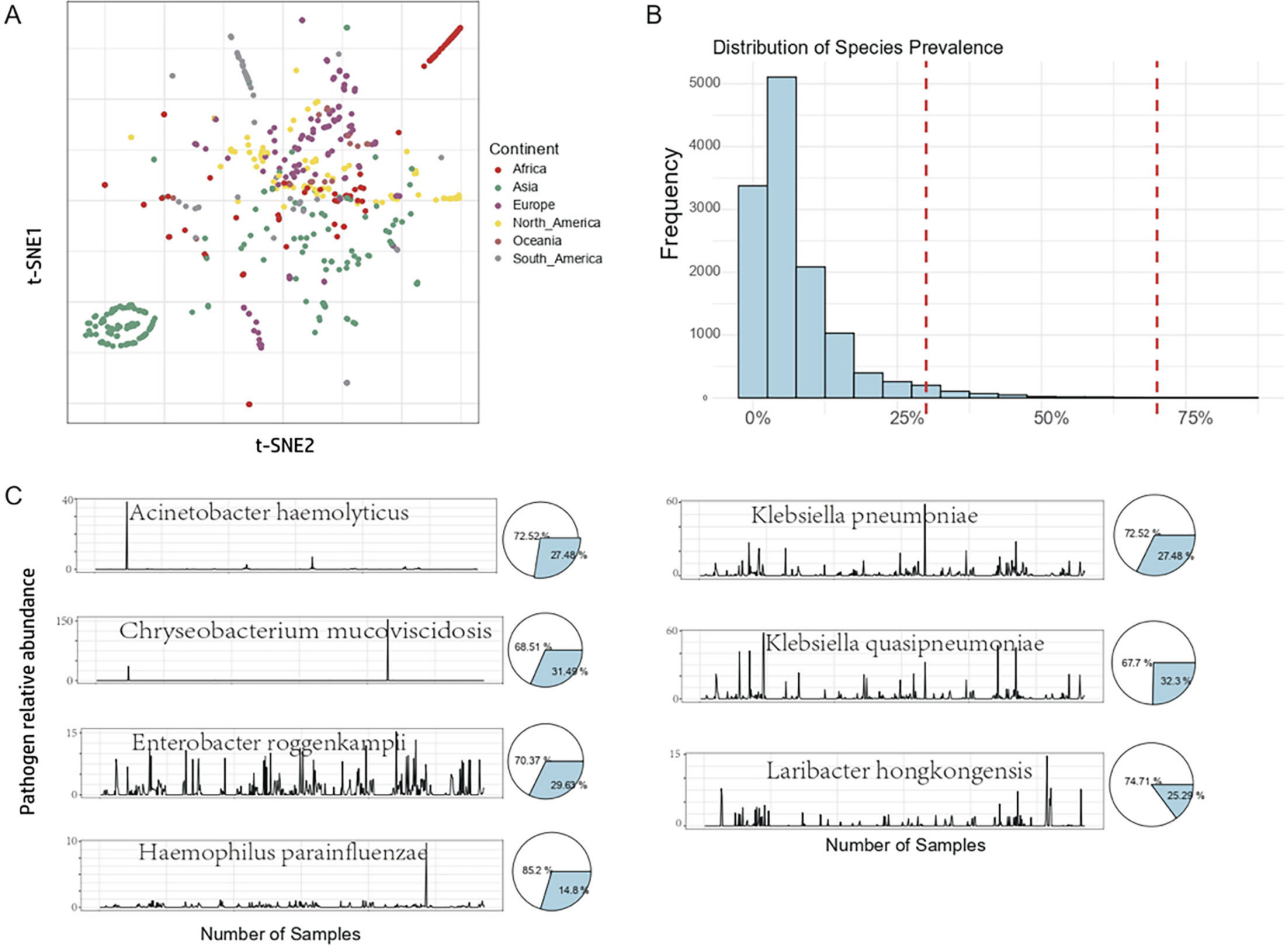
Community composition and environmental factors of microbial communities in global wastewater. (**A**) The t-SNE algorithm was employed to cluster the samples, and distinct sample clusters are depicted using different colors. (**B**) Distribution of species prevalence. The bar chart shows the distribution of species prevalence in samples, with the red dashed line indicating the prevalence threshold. (**C**) The pie chart on the right displays the proportions of prevalence. In each subplot, the bar chart indicates the relative abundances of samples in which the bacterial species was detected, and the pie chart illustrates the overall prevalence of the species across all samples, defined as the proportion of samples with at least one occurrence of the species.

We investigated distribution patterns of microbial species across global WWTP environments. T-distributed stochastic neighbor embedding (t-SNE) analysis revealed substantial heterogeneity in microbial communities across geographical regions. However, significant overlap among data points indicated the presence of common microbial species characteristics across diverse WWTP environments worldwide (Fig. 2A). Further examination of microbial species distribution in urban environments demonstrated that most species exhibited low frequencies, with prevalence values generally ranging from 1 to 25% (Fig. 2B). As prevalence increased, species numbers declined, creating a characteristic long-tailed distribution pattern.

In our analysis of wastewater samples from multiple cities, we identified a core community of microbial species present in more than 70% of samples. Among these, Proteobacteria was the most prevalent phylum, with *Hydrogenophaga pseudoflava* nearly ubiquitous across all samples. *H. pseudoflava*, a hydrogenotrophic denitrifier, exemplifies one of several species within the *Hydrogenophaga* genus exhibiting this trait (48). Additionally, prevalent strains, including *Bifidobacterium pseudocatenulatum* and *Bifidobacterium breve*, were detected. These bifidobacteria are well-established indicators of fecal contamination in humans and animals (49).

Non-core microorganisms exhibited greater standard variability, as indicated by higher standard deviations and kurtosis (Fig. 2C). Among these, several pathogens showed trends toward global dissemination. For example, *Acinetobacter haemolyticus* was present in fewer than 72% of the analyzed samples but showed notably higher prevalence in those collected from Kenya. This genus is associated with a range of clinical infections, including endocarditis, bacteremia, and community-acquired infections resulting from cross-infections among patients (50). The higher abundance of *A. haemolyticus* in certain geographic regions suggests that its prevalence may be influenced by local factors. Similarly, *Laribacter hongkongensis*, an emerging pathogen initially discovered in Hong Kong, is responsible for infectious diarrhea (51). Our analysis indicates a trend toward global spread, with increasing incidence rates in countries, such as Singapore, Spain, and Malta.

### Microbial and functional wastewater fingerprint profiles

**Fig 3:**
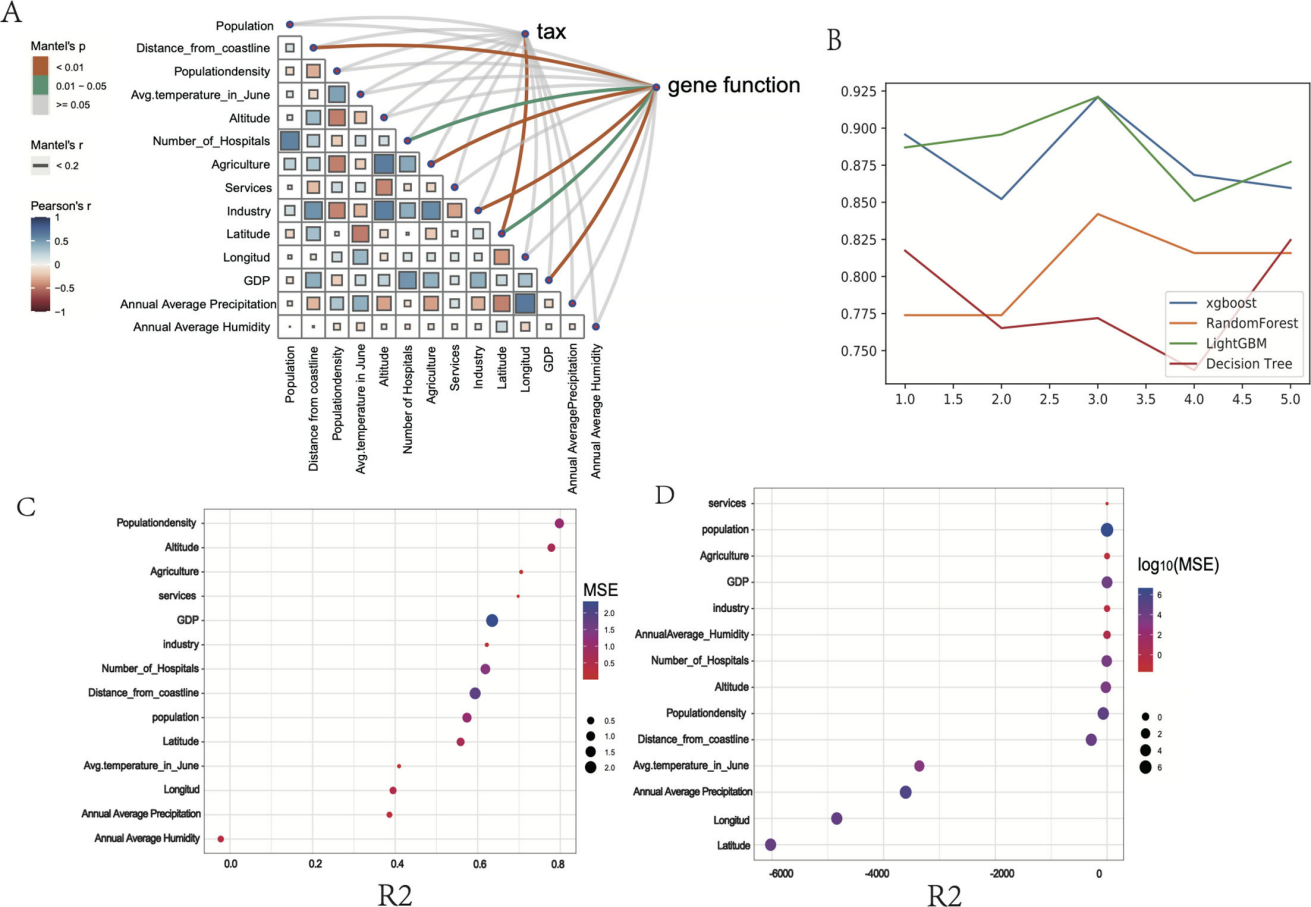
Microbial signatures in the environment of microbial communities in global wastewater. (**A**) Pairwise comparisons of environmental factors. The color gradients and box sizes represent Pearson’s correlation coefficient, with red indicating a positive correlation and blue indicating a negative correlation. Taxonomic and functional community compositions are assessed for their relationship with each environmental factor using Mantel tests. The width of the lines corresponds to the Mantel’s *r* statistic for the respective distance correlations. The model algorithm selection was based on five sets of cross-validation. (**B**) ROC curve of the RF model. The horizontal coordinate of this curve is the false-positive rate (FPR), while the vertical coordinate is the true-positive rate (TPR). (**C, D**) Predicting city characteristics based on gene functions (**C**) and species (**D**) through logistic regression. The *x*-axis represents *R*2 in logistic regression, while the size and color of the points represent MSE. *R*2 is used to measure the degree to which the model explains the variance in the observed data. *R*2 ranges from 0 to 1, where 0 indicates that the model cannot explain the variance of the dependent variable, and one indicates that the model completely explains the variance of the dependent variable. The mean squared error (MSE) is a metric used to measure the difference between the predicted and actual observed values in a model. It calculates the squared differences between the predicted and true values and takes the average of all the differences. A smaller MSE value indicates a smaller difference between the model’s predicted results and the actual observed values, indicating a better fit of the model.

To elucidate how environmental and socioeconomic factors influence WWTP microbial communities, we analyzed community structure and function. We assessed relationships between wastewater microbiome composition and function and various socioenvironmental factors using Mantel tests. Our analysis identified significant socioeconomic and geographical factors influencing wastewater microbiome composition, including latitude, longitude, distance from coastlines, gross domestic product, and agricultural and industrial activities. While microbial distribution was primarily influenced by latitude, community functions were affected by multiple environmental and socioeconomic variables. This suggests that despite variations in microbial taxonomic diversity, functional pathways remain conserved across continents, indicating consistent core microbial functions, regardless of geographic location (Fig. 3A).

Further analysis revealed that wastewater samples could be classified by continent based on functional and taxonomic characteristics. We developed a model using LightGBM with k-fold cross-validation to predict continental origin for each wastewater sample based on functional and taxonomic features (Fig. 3B). However, at the whole-community level, regression models relating taxonomic group abundance to environmental variables showed relatively low *R*² values, indicating that microbial communities were predominantly influenced by core metabolic pathways adapted to specific geochemical conditions (Fig. 3C and D). These pathways have diversified over time, with microbial clades competing within distinct ecological niches (52). This competition has generated extensive microbial diversity and significant functional redundancy across different wastewater systems.

### Transport proteins are pivotal to the WWTP functional network

**Fig 4:**
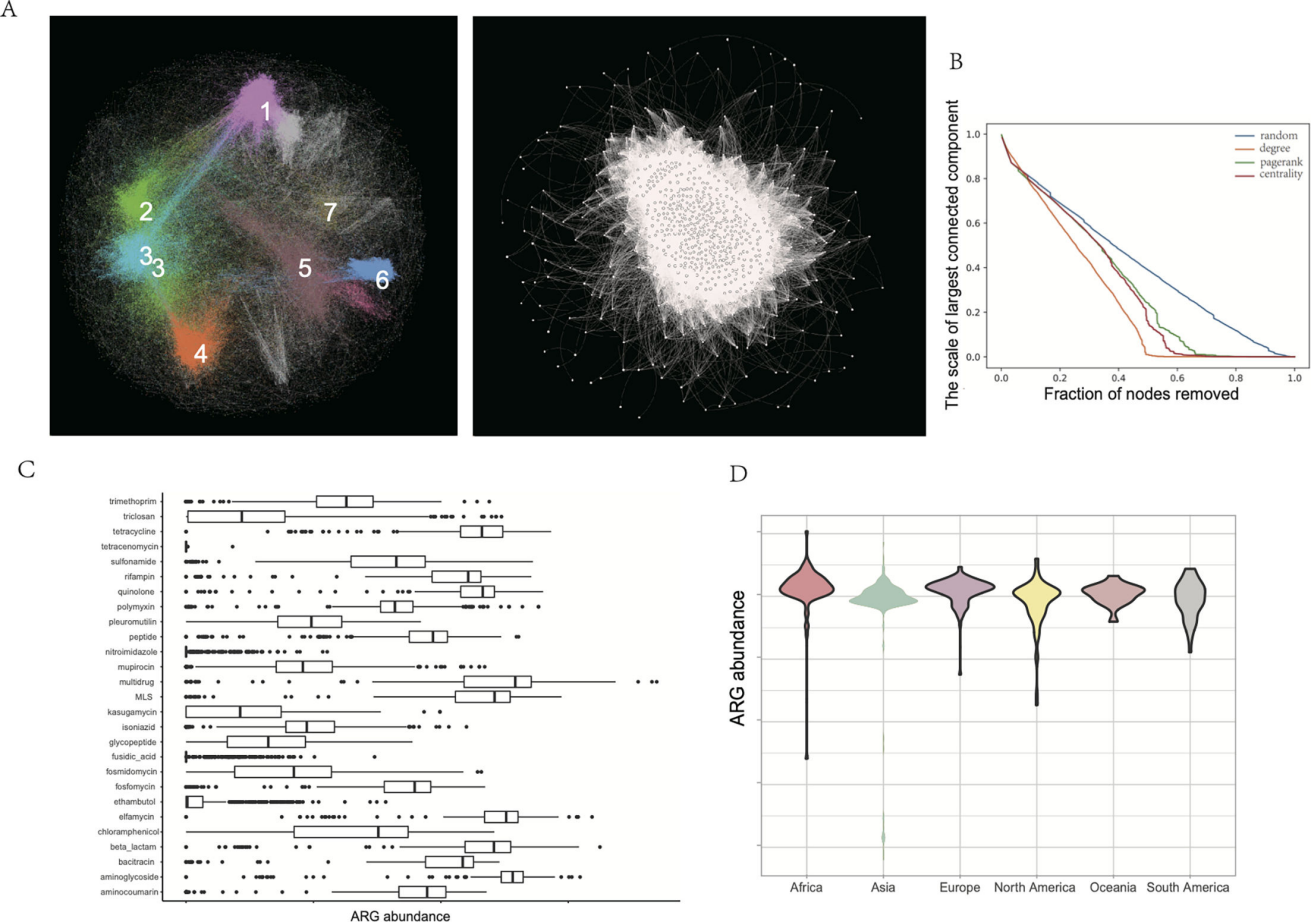
Microbial gene function of microbial communities in global wastewater. (**A**) The global genetic correlation network, encompassing all KEGG functions was constructed from the genetic correlation matrix. Gene pairs with Spearman coefficients greater than 0.6 were linked and visualized using a force-directed layout algorithm. Genes with higher correlation coefficients are mapped closer together, while those with lower coefficients are positioned further apart. Modules (M1–M10) are distinguished by different colors. The right side is a simplified network diagram highlighting the connections between nodes. (**B**) The graph illustrates the change in network connectivity as nodes are removed, showing the scale of the largest connected component as a function of the fraction of nodes removed. This method is used to assess the importance of individual nodes in maintaining network stability. (**C**) ARG gene abundance distribution box plot. This chart illustrates the distribution of abundance across various taxonomic units, with the *x*-axis depicting the logarithmic value of abundance [log(1 + abundance)] and the *y*-axis representing different taxonomic units. (**D**) ARG gene abundance violin plot across different regions.

We constructed a comprehensive functional network of the WWTP microbiome using 10,596 Kyoto Encyclopedia of Genes and Genomes (KEGG) Orthology genes expressed by wastewater microorganisms. This network comprises 8,638 functional nodes and 251,682 links (Fig. 4A). The dense interconnectivity suggests high integration, where inactivation of a single node could precipitate cascading effects beyond immediate neighbors.

In wastewater systems, microbial functional networks face constant disturbances. To understand their stability and resilience, we modeled node loss by simulating the removal of specific microbial functions. This approach illustrates the scale of the largest connected network component as a function of the fraction of nodes removed (Fig. 4B). This disruption propagation model, analogous to those used in power grid analysis, evaluates the impact of node inactivation on the overall network functionality (53). By comparing network efficiency before and after node disruption, we calculated the overall efficiency of each node and examined resulting connectivity changes. Similar to transportation and communication networks, high-degree nodes—those with extensive connectivity—demonstrated greater influence on network functionality. High-degree nodes were predominantly located in cluster 1, highlighting their importance in key metabolic pathways. Among significant nodes, transport proteins proved particularly crucial for network functionality. These proteins serve vital roles in nutrient uptake and energy metabolism within WWTPs. Specifically, amino acid transport proteins are essential for ammonia removal and nitrification processes, while carbohydrate transport proteins facilitate microbial carbon metabolism. Metal transport proteins, including those for iron, zinc, and manganese, participate in denitrification and help microbes adapt to heavy metal pollution. Additionally, multidrug-resistant transport proteins enable microbes to expel antibiotics, enhancing wastewater microbial network resilience against environmental stressors.

### Global distribution of antibiotic resistance genes in WWTP

We identified more than 70,000 antibiotic resistance genes (ARGs) in 11,919 genomes derived from WWTPs, encompassing 715 distinct subtypes (Fig. 4C). These ARGs span 28 drug classes and four major resistance mechanisms. The most prevalent ARGs conferred resistance to macrolides, tetracyclines, aminoglycosides, β-lactams, and sulfonamides. ARG numbers (Fig. 4D) and abundance varied substantially across cities, creating a highly diverse and uneven global distribution pattern. Resistance gene abundance showed significant inter-country variation. For instance, multidrug resistance genes exhibited an exceptionally high standard deviation of 810.81, reflecting considerable variability across regions. Conversely, genes conferring resistance to ethambutol (standard deviation = 3.57) and nitroimidazole (standard deviation = 1.19) showed relatively consistent abundance across countries. Countries with elevated ARG abundance span multiple continents, including African nations (e.g., Togo and Ghana) and Asian countries (e.g., Cambodia), whereas low ARG abundance was primarily concentrated in European nations (e.g., Switzerland, Germany, and Hungary) and North America (e.g., Canada).

### Metagenomic analysis reveals high diversity of viral population

**Fig 5:**
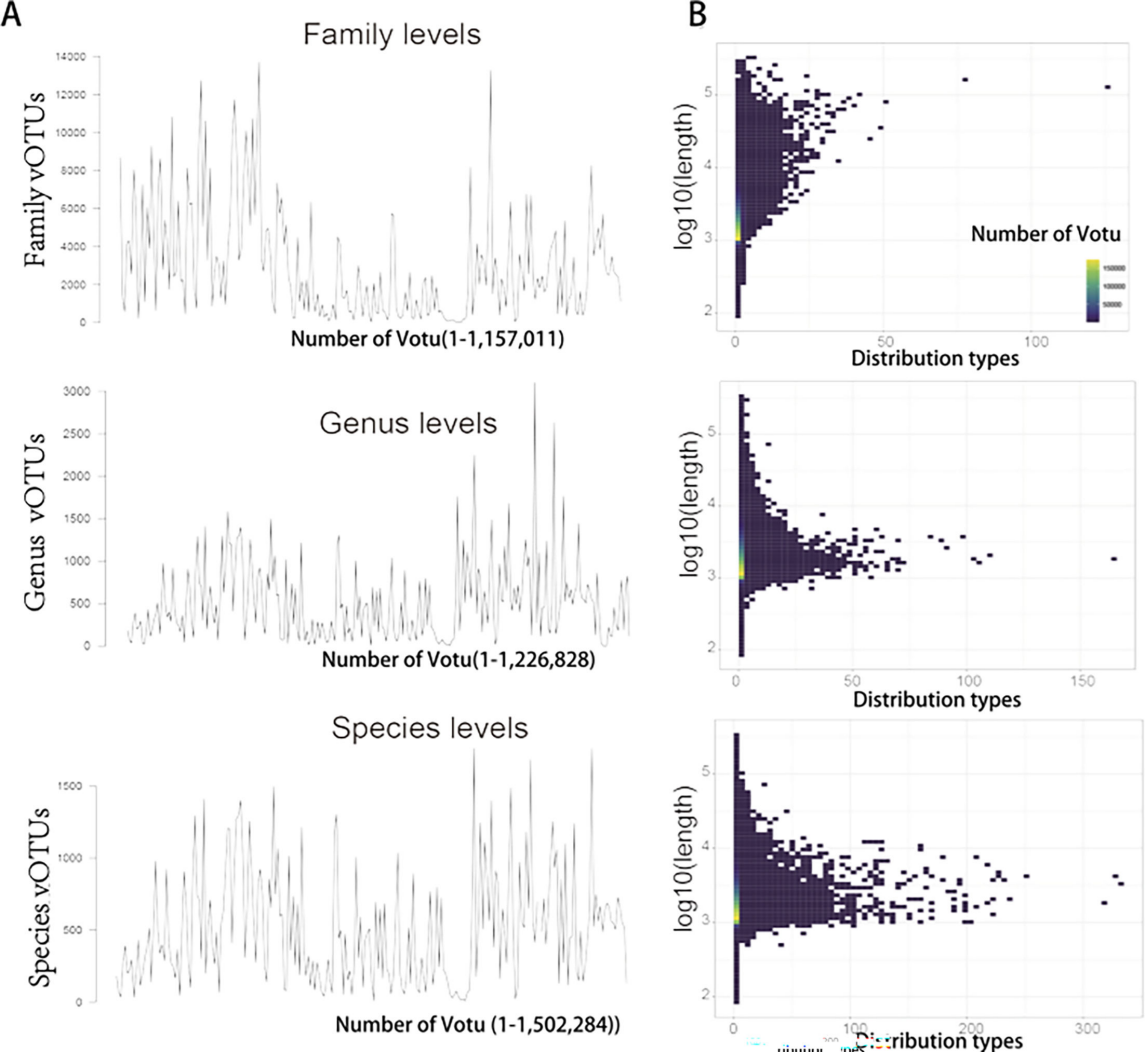
Overview of the viral catalog of global wastewater. (**A**) Accumulation curves for vOTUs from the viral catalog. Viral genomes were clustered into vOTUs at the species, genus, and family levels. (**B**) Logarithmic distribution of VOTU lengths. The graph displays the logarithmic distribution of VOTU lengths for different distribution types, with color representing the number of VOTUs.

To investigate wastewater viral populations, we conducted extensive metagenomic analysis of viral sequences from wastewater samples. The completeness of assembled viral genomes varied significantly, ranging from short fragments to nearly complete genomes. Using CheckV (54), we identified 1,732,482 genomes with at least 50% completeness. To quantify the viral genome diversity in the WWTP Metagenomic Viral Genomes catalog, we applied minimum information about an uncultivated virus genome (MIUViG) recommended criteria to define viral operational taxonomic units (vOTUs) at the species level (55). In total, we identified 1,502,284 vOTUs (Fig. 5A) with an average genome length of 2,363 bp. We further classified these genomes into genus- and family-level groups based on pairwise amino acid identity and gene sharing, resulting in 1,226,828 genus-level vOTUs and 1,157,011 family-level vOTUs (Fig. 5B). Our findings revealed exceptionally high wastewater virus diversity, with numerous distinct viruses identified at both family and genus levels, highlighting significant variation and evolutionary complexity within these higher taxonomic ranks.

### Functional diversity of wastewater viruses

**Fig 6:**
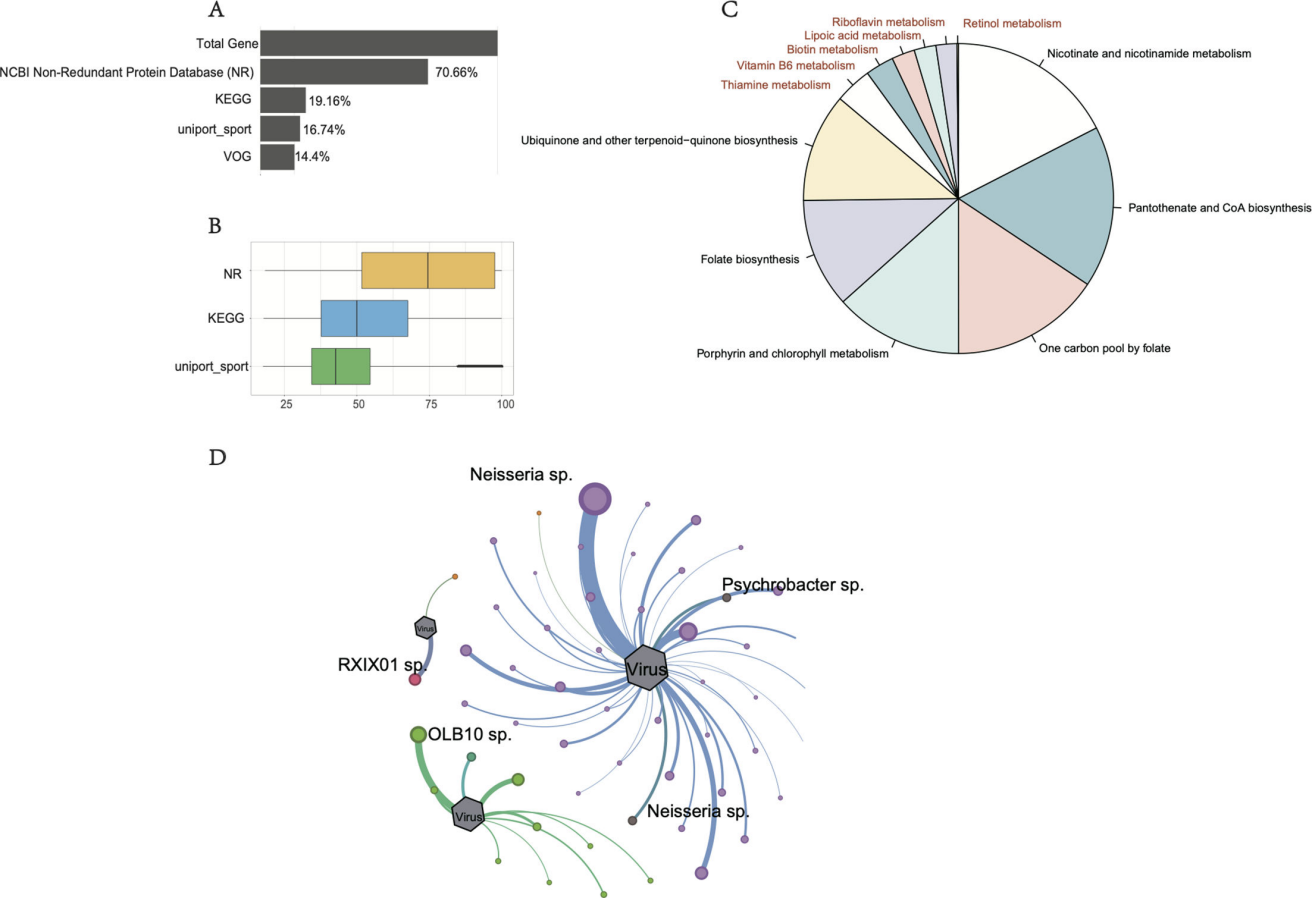
Functional capacity of the virome in global wastewater. (**A**) Protein-coding viral genes were identified across all vOTUs and compared with those that could be annotated from five databases, with the percentage of annotated genes calculated. (**B**) Maximum percentage identity of genes against different databases. (**C**) Proportion of metabolism functions shows the proportion of different metabolic pathways in the virome. (**D**) The network visualization illustrates the relationship between viruses and their bacterial hosts. Nodes represent individual bacteria and viruses, while edges indicate that a virus can infect certain bacteria. The color of the nodes signifies different taxonomic classifications. If a vOTU node is connected to different host nodes, it suggests that the predicted vOTU is likely to link to various host categories.

In our global wastewater virome study, we employed metaProdigal to predict open reading frames (ORFs) from sequence data. The annotation of 6,999,675 predicted ORFs revealed that approximately 20% of viral genes had no similar sequences in existing databases (Fig. 6A), suggesting these viruses harbor diverse unknown or unique proteins (Fig. 6B). We further explored the functional diversity of these viral genomes by analyzing them through the Virus Orthologous Groups (VOG) database. The hidden Markov model analysis indicated that the majority of viral functions were related to essential processes, such as the structural composition of viral particles, packaging, lysis, replication, and transcriptional regulation, all of which are crucial for the viral lifecycle and host-virus interactions.

To investigate auxiliary metabolic gene (AMG) roles in wastewater viruses, we focused on genes involved in cofactor and vitamin metabolism. This analysis revealed that AMGs were relatively rare, comprising only 3.2% of total functional genes (Fig. 6C). These pathways included thiamine, biotin, riboflavin, vitamin B6, and cobalamin biosynthesis, suggesting these viruses may influence microbial metabolic pathways in wastewater. We assessed virus-host interactions by analyzing CRISPR spacer sequences matching viral genomes. We identified 33,075 CRISPR spacer regions, of which 18.94% were significantly associated with viral genomes. Compared to other viruses, those carrying complex MCoV-AMGs demonstrated greater host specificity, particularly in common wastewater treatment facility microorganisms (Fig. 6D).

## DISCUSSION

### Impact of human activities on microbial communities in wastewater

Human activities have a significant influence on microbial communities in wastewater treatment plants (WWTPs), fundamentally shaping their diversity and functionality (56, 57). This comprehensive analysis of wastewater samples from 575 locations across 74 cities in 60 countries represents diverse climatic, geographical, and socioeconomic conditions. Previous research has demonstrated that human activities, such as urbanization, industrialization, and agriculture, substantially impact microbial and viral compositions, leading to significant regional variations (57, 58). Notably, wastewater from industrialized regions exhibits elevated concentrations of antibiotic resistance genes (ARGs), reflecting the selective pressures imposed by industrial effluents on microbial communities (59, 60). This study highlights the high resilience of WWTP networks, which maintain stability despite fluctuations in pollutant loads and microbial dynamics. These systems demonstrate their adaptive capacity by adjusting their microbial composition in response to environmental pressures, thus maintaining functional integrity (61, 62).

### Biogeographic distribution of wastewater microbial communities

Our analysis reveals significant geographical variations in microbial composition and functionality. Notably, microbial communities in tropical wastewater exhibit marked differences compared to those in temperate regions (63). These variations highlight the combined influence of climatic conditions, geographic location, and human activities on microbial communities in tropical environments (64). Through an in-depth analysis of wastewater samples from multiple cities, we observed that over 70% of the samples contained a stable core microbial community. This finding indicates that despite differences in environmental conditions and human activities across various regions worldwide, certain microbial communities possess a universal functionality. Among these core microbial communities, bacteria from the *Proteobacteria phylum* stand out, especially *Hydrogenophaga pseudoflava*, which is present in almost all samples. As a hydrogenotrophic denitrifier, it contributes to the conversion of nitrates into nitrogen gas, playing a role in the biological nitrogen removal process in sewage. Additionally, other species within the *Hydrogenophaga* genus exhibit similar functional characteristics, further emphasizing the importance of these microorganisms in ecosystems. We also identified prevalent strains, such as *Bifidobacterium pseudolongum* and *B. breve*, which are often considered biological indicators of human and animal fecal contamination. The geographical coordinates of sampling sites provide insights into climatic conditions, environmental settings, and local biodiversity, all of which contribute to shaping the diversity and structure of microbial life in wastewater (65). The observed correlations between specific microbial profiles and environmental and socioeconomic parameters suggest a transition from a reactive to a predictive approach in wastewater management (66). By identifying microbial signatures associated with specific factors, it becomes possible to design WWTPs that address the unique challenges of their urban environments, ultimately enhancing the resilience and sustainability of urban ecosystems (67).

Regarding the global distribution of antibiotic resistance genes, our analysis revealed that countries with elevated ARG abundance span multiple continents, including African nations (e.g., Togo and Ghana) and Asian countries (e.g., Cambodia). Conversely, regions with lower ARG prevalence are predominantly concentrated in Europe and North America. These geographic disparities likely reflect complex interactions between antibiotic usage patterns, healthcare infrastructure, and agricultural practices unique to each region. Particularly concerning is the situation in certain low-income countries, where inadequate regulation of antibiotic consumption may contribute to elevated ARG abundance, potentially increasing transmission risks of antibiotic-resistant microorganisms to human populations.

### Global perspectives on microbial resistance and the role of viruses

The COVID-19 pandemic has emphasized the interconnectedness of global communities and highlighted the necessity for a unified global approach to microbial monitoring within WWTPs (1, 68). The variations in ARG prevalence observed across different urban environments underscore the complex interplay between antibiotic consumption habits, local environmental factors, and unique regional microbial ecosystems. Notably, the lower prevalence of ARGs in developed countries may be attributed to regulated antibiotic use and the implementation of advanced wastewater treatment technologies (69). In this era of unprecedented human and material mobility, microbial threats have become pervasive and intangible. The cross-border movement of individuals and goods has inadvertently facilitated the spread of foreign microorganisms, complicating the control of infectious diseases. The case of *Laribacter hongkongensis*, once geographically confined, now requires global attention, highlighting the imperative for international collaboration and vigilance in public health (70).

### Ecological role of viruses in wastewater treatment systems

The recognition of viruses as essential components of the wastewater ecosystem signifies a fundamental shift in our understanding of microbial dynamics within these intricate systems. The in-depth analysis of viral genomes in this study revealed an extensive diversity, with the majority comprising previously uncharacterized viruses (71). This diversity enriches our understanding of viral roles in microbial ecosystems and their potential for effective monitoring and management. Despite being frequently overlooked in wastewater treatment, viruses are key determinants of microbial community composition and function (72). Viruses not only directly affect the growth and function of host microorganisms through lytic lysis or alterations in host gene expression but also deeply integrate into the host’s metabolic networks through auxiliary metabolic genes (AMGs) (72–74). In our functional analysis, we particularly focused on virus-encoded genes involved in coenzyme and vitamin metabolism. While these genes account for only 3.2% of the total functional genes in viral genomes, the metabolic pathways they participate in, such as thiamine metabolism, biotin metabolism, and riboflavin metabolism, suggest that viruses regulate host metabolic activities from the bottom up through these genes. As "metabolic regulators," viruses play a crucial role in the functional integration of microbial communities, controlling host energy metabolism and thereby influencing the stability and diversity of microbial communities. Furthermore, viruses may enhance the biosynthesis of their components by exploiting host metabolic processes, thereby promoting viral replication and propagation. The auxiliary metabolic genes carried by viruses enable them to more efficiently utilize host resources, driving host metabolic networks to support viral replication.

### Wastewater as a reflection of global human activity

Global studies on wastewater microbial and viral genomes elucidate the significant impact of human societies on wastewater treatment systems and demonstrate how social characteristics are reflected in the microbial composition of wastewater (75). Microbial communities in wastewater exhibit distinct local characteristics, while pathogens and ARGs show greater tendencies for global dissemination. These wastewater microbiomes play a crucial role in maintaining system stability through extensive transporter networks, facilitating adaptation to various environmental challenges. Viruses in these microbial communities infect host microorganisms and encode auxiliary metabolic genes, driving metabolic enhancement and diversification. These findings not only deepen our understanding of wastewater microbiology but also provide critical insights for improving wastewater management and environmental health practices. Additionally, they highlight the urgent need for more stringent antibiotic regulation to mitigate the spread of ARGs (76). Analysis of wastewater microbial characteristics significantly deepens our understanding of their complexity and diversity, providing valuable guidance for the design and operation of wastewater treatment plants (77). This study highlights the intricate relationship between human activities and the microbial and viral ecosystems in wastewater, revealing the interconnectedness of societal factors, environmental conditions, and microbial dynamics. As global challenges, such as antimicrobial resistance and emerging pathogens, intensify, wastewater surveillance emerges as a valuable early warning system, providing real-time insights into public health trends. These findings highlight the transformative potential of wastewater-based epidemiology, not only as a critical tool for environmental monitoring but also as a foundation for sustainable urban development and public health resilience.

### Conclusions

This study provides a comprehensive global perspective on wastewater microbial and viral genomes, focusing on quantifying the distribution, types, and dynamics of environmental microbiomes. The analysis illuminates the intricate interactions within microbial and viral communities in WWTPs worldwide, emphasizing the significant influence of human activities on these ecosystems. An examination of microbial genomes from 575 metagenomes reveals that urbanization, industrialization, and agriculture significantly shape regional variations in microbial composition and function. These findings demonstrate how environmental and socioeconomic factors, such as geography and antibiotic usage, shape microbial profiles in wastewater and influence the dissemination of pathogens and ARGs. The study provides valuable insights for improving wastewater management and public health strategies. Viruses often overlooked in wastewater ecosystems play a crucial role in regulating microbial communities and facilitating gene transfer, thereby influencing ecosystem functions and metabolic pathways. The global proliferation of ARGs observed in this research emphasizes the urgent need for more stringent antibiotic regulations and improved microbial monitoring to mitigate public health risks (78).

## Data Availability

All scripts for this article are available at https://github.com/alienn233/WWTP. All data used in this study were obtained from publicly available sources, with complete accession numbers and metadata provided in Table S2.

## SUPPLEMENTAL MATERIAL

Graphical Abstract msystems.01428-24-s0001.pdf Illustration of a large-scale project involving the collection and analysis of wastewater samples from diverse geographical regions.

Supplemental Tables msystems.01428-24-s0002.xlsx Tables S1 and S2.

ASM does not own the copyrights to Supplemental Material that may be linked to, or accessed through, an article. The authors have granted ASM a non-exclusive, world-wide license to publish the Supplemental Material files. Please contact the corresponding author directly for reuse.
