# Supplementary material for: Global wastewater microbiome reveals core bacterial community and viral diversity with regional antibiotic resistance patterns
Source: mSystems. 2025 Sep 17;10(10):e01428-24. doi: 10.1128/msystems.01428-24 (PMC12542734; doi:10.1128/msystems.01428-24)
Supplement: Graphical Abstract — Illustration of a large-scale project involving the collection and analysis of wastewater samples from diverse geographical regions. [file msystems.01428-24-s0001.pdf]

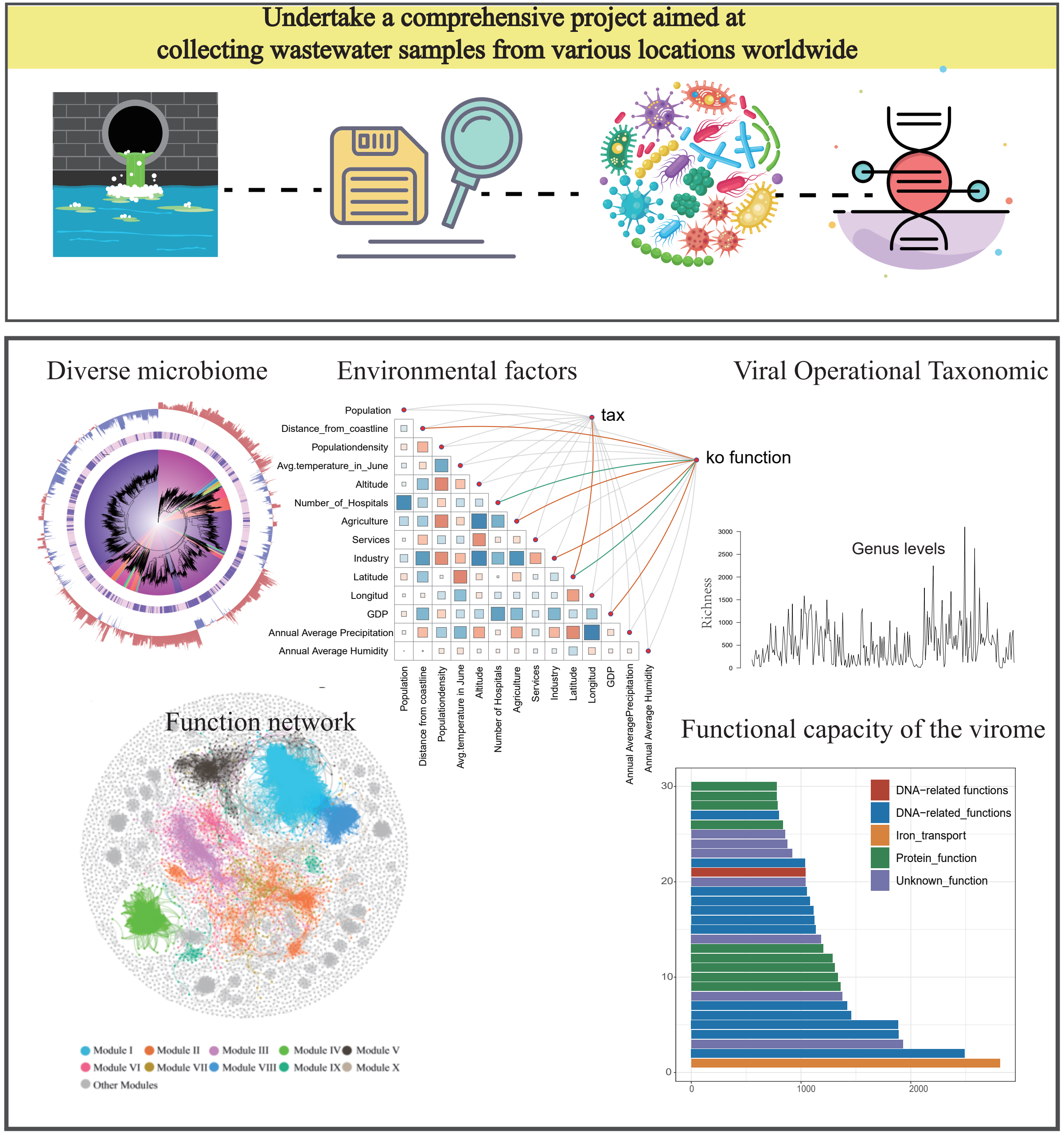
Undertake a comprehensive project aimed at
collecting wastewater samples from various locations worldwide
Diverse microbiome
Environmental factors
Viral Operational Taxonomic
tax
ko function
Population
Distance_from_coastline
Populationdensity
Avg.temperature_in_June
Altitude
Number_of_Hospitals
Agriculture
Services
Industry
Latitude
Longitud
GDP
Annual Average Precipitation
Annual Average Humidity
Distance from coastline
Avg.temperature in June
Number of Hospitals
Annual AveragePrecipitation
Genus levels
Richness
Function network
Module I
Module II
Module III
Module IV
Module V
Module VI
Module VII
Module VIII
Module IX
Module X
Other Modules
Functional capacity of the virome
DNA−related functions
DNA−related_functions
Iron_transport
Protein_function
Unknown_function
